# Renal involvement in a patient with the chronic visceral subtype of acid sphingomyelinase deficiency resembles Fabry disease

**DOI:** 10.1002/jmd2.12242

**Published:** 2021-07-26

**Authors:** Eline C. B. Eskes, Martijn J. C. van der Lienden, Joris J. T. H. Roelofs, Liffert Vogt, Johannes M. F. G. Aerts, Jan Aten, Carla E. M. Hollak

**Affiliations:** ^1^ Department of Endocrinology and Metabolism Amsterdam UMC, University of Amsterdam Amsterdam The Netherlands; ^2^ Department of Pathology Amsterdam UMC, University of Amsterdam Amsterdam The Netherlands; ^3^ Amsterdam UMC, Amsterdam Cardiovascular Sciences Department of Internal Medicine, section Nephrology, University of Amsterdam Amsterdam The Netherlands; ^4^ Leiden Institute of Chemistry, Department of Medical Biochemistry University of Leiden Leiden The Netherlands

**Keywords:** acid sphingomyelinase deficiency, Fabry disease, Gaucher disease, histopathology, Niemann‐Pick disease, renal manifestations

## Abstract

Acid sphingomyelinase deficiency (ASMD) is a lysosomal storage disease (LSD) in which sphingomyelin accumulates due to deficient acid sphingomyelinase. In the chronic visceral subtype, organ manifestations are generally limited to the spleen, liver, and lungs. We report a male patient with the chronic visceral subtype who developed proteinuria and renal insufficiency at the age of 49. In renal tissue, foam cells were observed in the glomeruli as well as sphingomyelin accumulation within podocytes, mesangial cells, endothelial cells, and tubular epithelial cells. Although macrophages are the primary storage cells in both ASMD and Gaucher disease, comparison to the histopathological findings in Gaucher and Fabry disease revealed a diffuse storage pattern in multiple renal cell types, closer resembling the pattern found in Fabry disease.

**Abbreviations**
ASM: acid sphingomyelinase
ASMD: acid sphingomyelinase deficiency
CCL18: chemokine C‐C motif ligand 18
CKD‐EPI: Chronic Kidney Disease Epidemiology Collaboration
eGFR: estimated glomerular filtration rate
ERT: enzyme replacement therapy
FEV1: forced expiratory volume
FVC: forced vital capacity
Gb3: globotriaosylceramide
HIV: human immunodeficiency virus
HRCT: high‐resolution computed tomography
LSD: lysosomal storage disease
MN: multiples of normal
MRI: magnetic resonance imaging
SD: standard deviation

## 1 INTRODUCTION

Acid sphingomyelinase deficiency (ASMD; OMIM 607616), formerly known as Niemann‐Pick disease types A and B, is a lysosomal storage disease (LSD) in which sphingomyelin accumulates due to deficiency of the enzyme acid sphingomyelinase (ASM; EC 3.1.4.12).^1^ The diagnosis can be made by demonstrating deficient enzyme activity in leukocytes or fibroblasts further supported by analysis of genetic variants in the *SMPD1* gene. ASMD is rare, with an estimated birth prevalence of approximately 1 in 200 000.^2^, ^3^ Although there is a spectrum of manifestations, traditionally three subtypes are distinguished: the infantile neurovisceral type, which is lethal before the age of three, the intermediate chronic neurovisceral type, in which patients survive into childhood, but are neurologically affected, and the chronic visceral type, which comprises a broad spectrum from severely affected adolescents to elderly adults with minimal disease, all without neurological symptoms. The most common visceral manifestations are hepatosplenomegaly and interstitial lung disease.^4^, ^5^, ^6^ Treatment is not available yet, so patients are monitored carefully and offered supportive care. Enzyme replacement therapy (ERT) (olipudase alfa TM, Sanofi, MA) is under investigation and has already shown promise in reducing splenomegaly and improving pulmonary function.^7^ Several cohort studies have recently been launched to better understand the clinical variability, natural disease course, and biomarker profile.^5^, ^8^, ^9^, ^10^ In the context of these studies, unexpected manifestations may become apparent, such as renal disease. Renal involvement in patients with ASMD is unusual and has rarely been reported, mainly as a coincidental finding during postmortem pathology studies.^11^, ^12^, ^13^, ^14^, ^15^ Here, we describe a patient who showed progressive proteinuria and decline of renal function, with signs of lysosomal storage as the most likely culprit.

## 2 CASE

**FIGURE 1:**
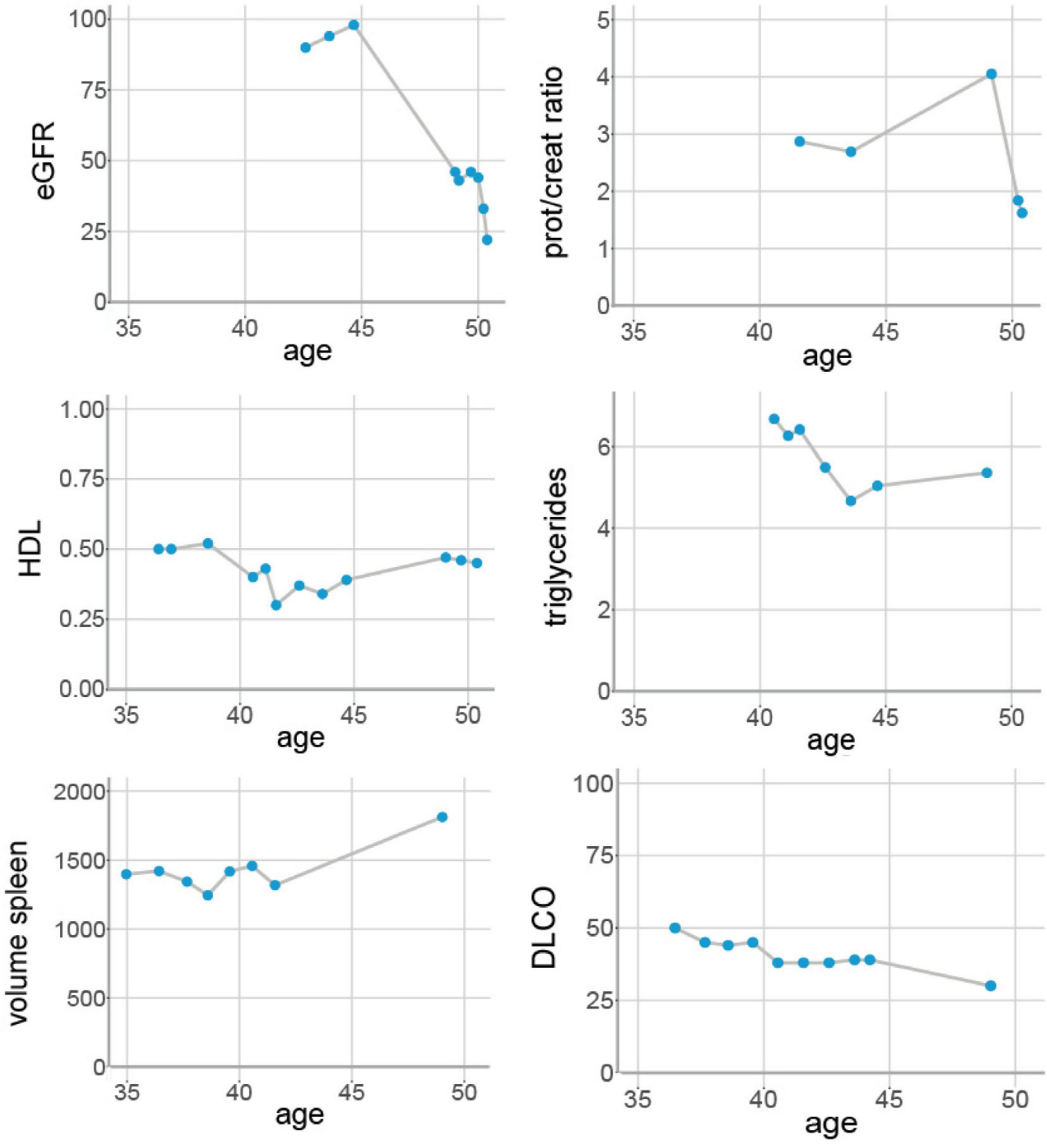
Clinical parameters and their course over time. eGFR (mL/min/1.73 m^2^), HDL (mmol/L) and triglycerides (mmol/L) are measured in plasma, prot/creat ratio (mg/g) in 24 hours urine, volume of the spleen (ml) is measured on MRI, DLCO (mmol/(min*kPa*L)) is measured in a body box. eGFR, estimated glomerular filtration rate; DLCO, diffusion capacity of the lung for carbon monoxide; HDL, high‐density lipoprotein; prot/creat ratio, ratio between protein and creatinine in 24 hours urine

The patient, a 35 year‐old male from Tunisia, was referred to the Amsterdam UMC in 2006 because of fatigue and hepatosplenomegaly. His medical history revealed hepatosplenomegaly and epistaxis during childhood. A genetic background was suspected since his sister displayed similar symptoms. A diagnosis of ASMD was suggested in the past and confirmed after referral to our center. Biochemical and genetic investigations showed an ASM activity in leukocytes of 2.4 nmol/mg/17 h (reference range 10‐53 nmol/mg/17 h) and homozygosity for the well‐known pathogenic p.Arg610del mutation in the *SMPD1* gene. Symptoms at the time were abdominal distension and progressive dyspnea with decreased exercise tolerance. Thorough investigations revealed a liver volume of 2893 mL (MN 2 [multiples of normal]^16^) and spleen volume of 1398 mL (MN 12.3) on magnetic resonance imaging (MRI). Pulmonary function tests showed near normal lung volumes (forced vital capacity [FVC] 82% of predicted and forced expiratory volume [FEV1] 75% of predicted), but severely compromised CO‐diffusion capacity (0.80 mmol/min/kPa/L [50% of predicted], corrected for alveolar volume). On a high‐resolution computed tomography (HRCT), typical findings of diffuse ground glass opacities and a reticular pattern were present. He was monitored in our outpatient clinic for the next 14 years at irregular intervals, with more or less stable parameters (Figure 1). At the age of 49, after an interval of 3 years, he reported complaints of polydipsia and polyuria. Normal glucose levels were established; however, an unexpected fall was found in estimated glomerular filtration rate (eGFR) to 46 mL/min (according to the formula as proposed by the Chronic Kidney Disease Epidemiology Collaboration [CKD‐EPI]), which was 98 mL/min 3 years earlier. ASMD‐related parameters showed progression: spleen volume increased (from 1320 mL to 1810 mL) and diffusion capacity decreased (from 39% to 30% of predicted). Chemokine C‐C motif ligand 18 (CCL18) values were similar (patient is deficient in chitotriosidase activity). Factors that might have evoked or contributed to renal function deterioration were investigated: the patient did not use any medication, he smoked approximately 10 cigarettes per day, his blood pressure was normal and his body mass index (BMI) was 18.5 kg/m^2^. The patient's family history was negative for renal diseases.

Urinanalysis showed nephrotic range proteinuria of 3.5 g/24 h (protein/creatinine ratio of 4049 mg/g) without signs of hematuria or leukocyturia. Immunological and virological work‐up (ie, plasma proteins including monoclonal proteins and serology for human immunodeficiency virus [HIV] and hepatitis types A, B, and C) did not reveal a cause for renal failure, thus a biopsy was performed.

## 3 RENAL BIOPSY

**FIGURE 2:**
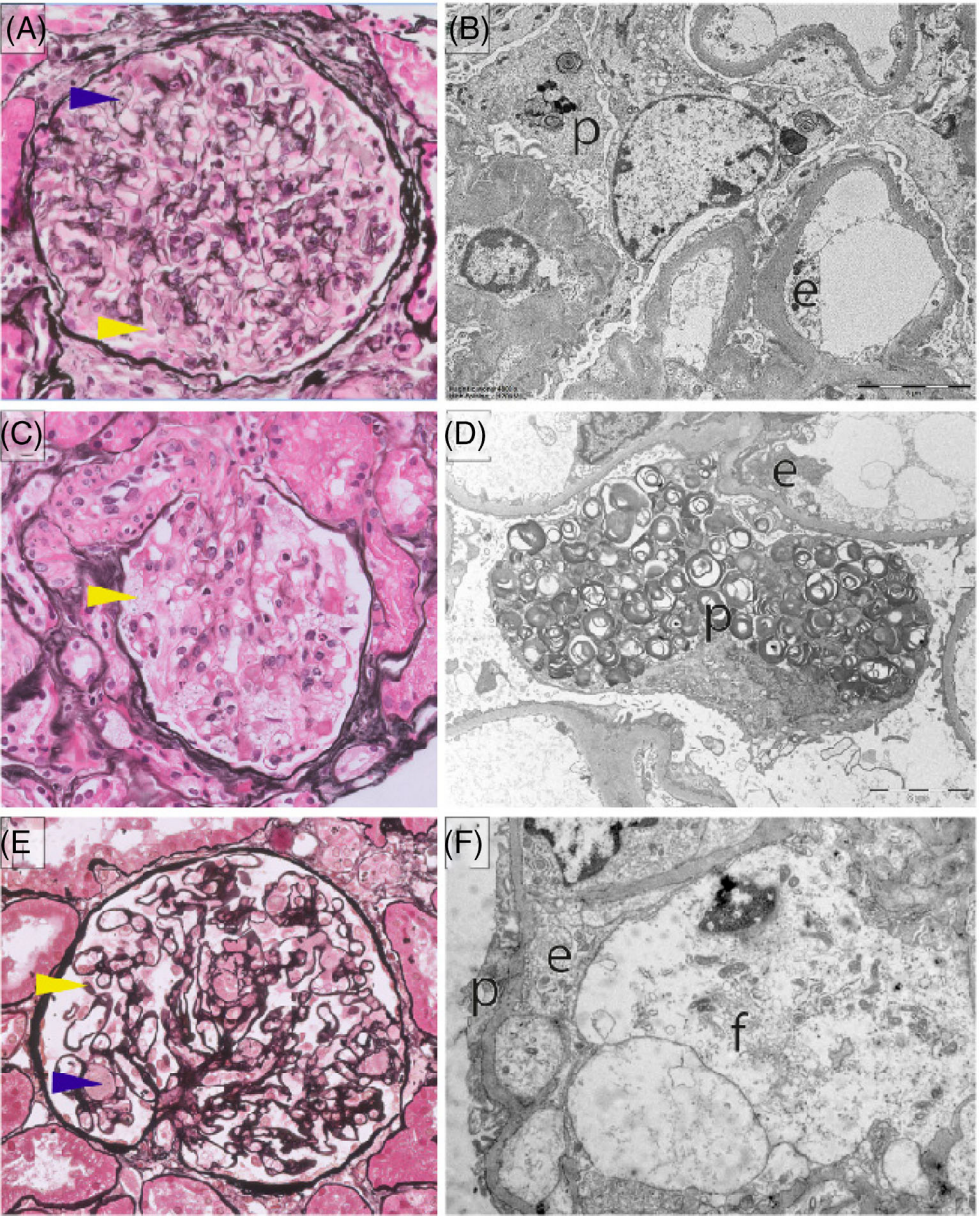
Light microscopy (Jones' methenamine silver staining; (A, C, E) and electron microscopy, (B, D, F) of glomeruli of ASMD, (A, B), Fabry, (C, D) and Gaucher, (E, F), patients. A, In ASMD, podocytes are not swollen and do not show vacuolization. A few endocapillary foam cells are present. B, Multilamellar deposits are present in podocytes. C, In Fabry, vacuolated podocytes are abundant. D, Podocytes contain many multilamellar bodies. E, In Gaucher, the glomerulus contains abundant endocapillary foam cells, podocytes appear normal. F, Lipid accumulation is limited to endocapillary foam cells. p: podocyte; e: endothelial cell; f: foam cell; yellow arrows: podocytes; blue arrows: foam cells

**FIGURE 3:**
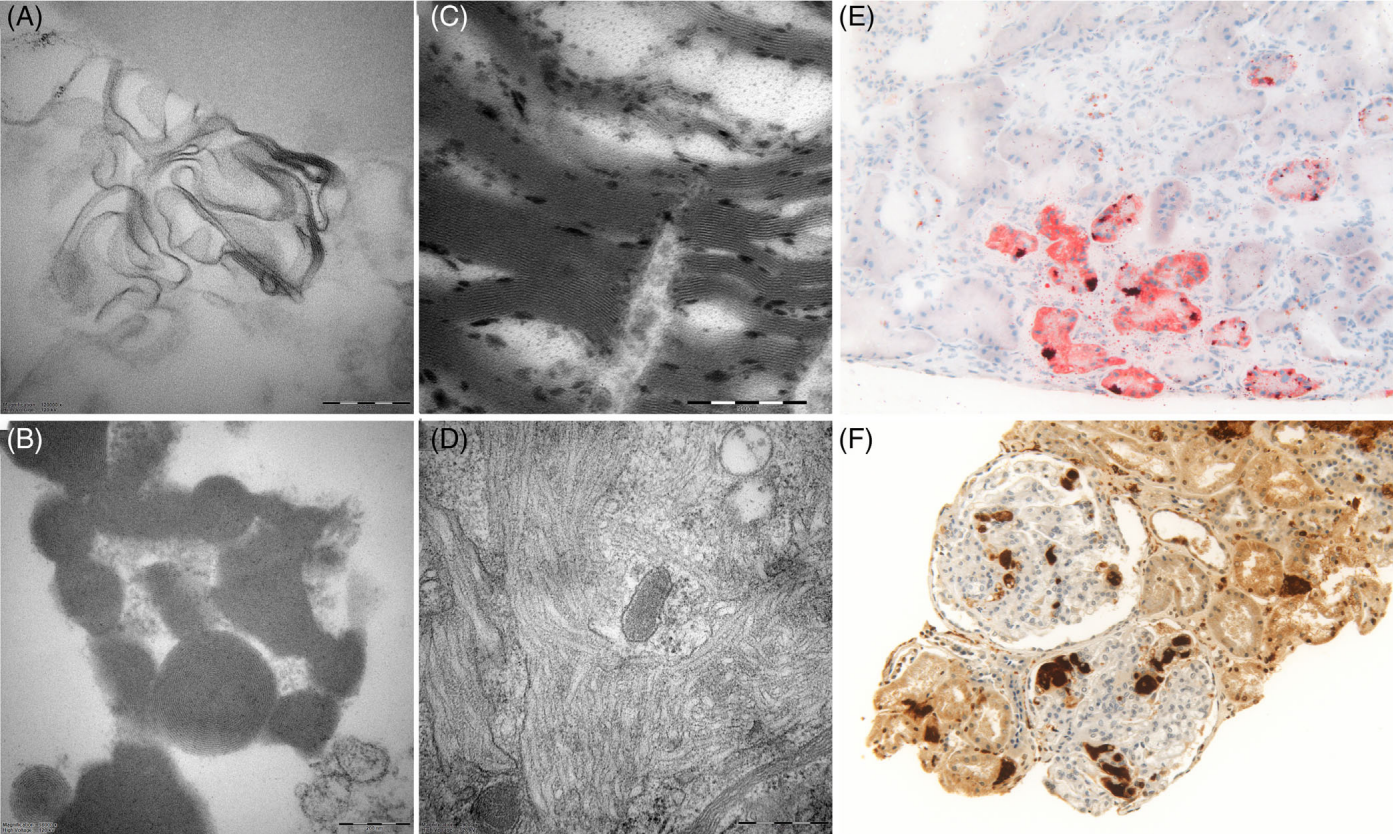
Electron microscopy, (A‐D) and light microscopy, (E, F) images of renal tissue of ASMD, Fabry and Gaucher patient. A, B, Multilamellar deposits present in ASMD podocytes. C, Multilamellar bodies present in Fabry podocytes. D, Storage material in glomerular Gaucher cells appears as microtubular structures, E, Oil‐Red O staining of ASMD kidney shows intracellular presence of neutral lipids in tubular epithelium (magnification ×20). F, CD68‐staining of a Gaucher kidney reveals glomerular and tubulointerstitial abundance of macrophages (magnification ×10)

Light microscopy showed a limited amount of renal cortical tissue, with only four glomeruli present in the paraffin sections, none of which were globally sclerosed. Renal parenchyma showed extensive chronic damage, consisting of interstitial fibrosis and tubular atrophy in approximately 70% of the biopsy, accompanied by a scanty resorption infiltrate. Glomeruli showed mild endocapillary hypercellularity and presence of a few foam cells within the capillary lumens (Figure 2A). Podocytes were unremarkable, without swelling or vacuolated appearance. An additional Oil‐Red‐O staining showed lipid accumulation within glomerular foam cells, tubular epithelial cells, and arteriolar endothelium (Figure 3E). Immune fluorescence showed no specific signal for immunoglobulins, complement or light chains. Electron microscopy revealed numerous lysosomal myelin figures, within podocytes (Figure 2B), glomerular macrophages, mesangial cells, endothelial cells, and tubular epithelial cells. There was moderate effacement of podocyte foot processes, considered secondary to the lipid storage. The storage material observed in podocytes (Figure 3A,B) has a periodicity of 5.7 nm (SD [SD] 0.5) as measured by two independent observers, deviating from its previously reported periodicity measured in other tissues of ASMD patients.^17^, ^18^

## 4 DISCUSSION

Renal involvement in ASMD has been reported in a few cases only.^11^, ^12^, ^13^, ^14^, ^15^ These patients were young, representing severe phenotypes of ASMD with extensive manifestations at a young age, opposed to our patient who has the chronic visceral phenotype with an attenuated course of disease. In three out of four cases, findings of lipid accumulation in the kidneys were established postmortem without a clinical history of kidney injury. The only case describing a patient with clinical manifestations concerns a young girl with severe manifestations of the chronic neurovisceral subtype of ASMD.^11^ This patient underwent splenectomy at the age of 15 months and received a liver transplant at the age of 4 years. Following a Ross procedure and mitral valve annuloplasty because of aortic insufficiency and moderate mitral insufficiency, renal function declined (whether there was proteinuria is not reported). A renal biopsy showed glomerulosclerosis, foam cells in the interstitium and vacuolated foamy podocytes with extensive effacement. Myelin‐like inclusions were found in tubular epithelial cells and endothelial cells of the peritubular capillaries. This is in line with findings in the renal tissue of ASM‐knock out mice, as reported by Kuemmel et al, who described sphingomyelin storage in these mice in glomerular and epithelial cells of the tubules, as well as in mesangial cells and podocytes.^19^

The existence of a genotype‐phenotype relation seems unlikely since our patient has the very common p.Arg610del mutation and reports of clinical renal disease in ASMD are scarce. Mutations are not reported in four out of five aforementioned cases, most of them were published before DNA analysis was part of standard practice.^11^, ^12^, ^13^, ^14^ The publication by Thurberg is the only one reporting a mutation; however, this publication concerns a child with the infantile neurovisceral subtype of ASMD.^15^

Renal involvement is rare in ASMD. Since the macrophage is the primary storage cell, similar to Gaucher disease (OMIM 230800), it was expected that the pathology resembled the pattern seen in the rare cases of renal involvement as described in Gaucher disease. Gaucher disease, caused by β‐glucosidase (EC 4.2.1.25) deficiency and resulting in storage of glucosylceramide, mainly in macrophages, shows clinical overlap with ASMD. In exceptional cases, renal involvement in Gaucher patients has been described.^20^, ^21^, ^22^, ^23^, ^24^, ^25^, ^26^, ^27^ However, we established that the pattern of storage in renal tissue differs between ASMD and Gaucher: in Gaucher cases foamy macrophages are observed in the glomeruli and the interstitium, while other renal cells are involved to a limited extent (Figures 2E,F and 3F), whereas in our ASMD patient, foam cells are observed in the glomeruli, but in addition lipid storage in podocytes, mesangial cells, endothelial cells, and tubular epithelial cells is found. Grafft et al report similar findings in the young ASMD patient.^11^

In fact, the storage pattern as found in these two ASMD cases resembles renal pathology as seen in Fabry disease (OMIM 301500), an X‐linked LSD with accumulation of globotriaosylceramide (Gb3) due to a deficiency of alpha‐galactosidase A (EC 3.2.1.22). Renal involvement in Fabry disease is common, specifically in classically affected males, manifesting as early proteinuria and loss of podocytes followed by renal failure between the third and fifth decade of life.^28^, ^29^ A histological hallmark of Fabry nephropathy is lipid storage in a variety of renal cells: podocytes, mesangial cells, endothelial cells of capillaries and arteries, distal tubule, vascular smooth muscle cells, and interstitial cells (Figure 2C,D).^28^, ^29^, ^30^, ^31^, ^32^

Although the macrophage is the primary storage cell in both ASMD and Gaucher, a more diffuse pattern of storage is observed in ASMD. Sphingomyelin is the most abundant sphingolipid in the cell and is a main component of the plasma membrane.^33^ Therefore, in ASMD, there is probably more storage of sphingomyelin outside the macrophages and the lysosome than is the case for glucosylceramide in Gaucher. This diffuse pattern of storage of sphingomyelin in ASMD might lead to different secondary pathophysiological processes. Moreover, it has been hypothesized that substrate accumulation in LSDs causes secondary lysosomal dysfunction resulting in accumulation of other sphingolipids.^34^ Increases in bioactive sphingolipids, such as sphingosine‐1‐phosphate, have been associated with kidney disease.^35^, ^36^, ^37^

Along with the periodicity of the storage material in the ASMD patient (ie, 5.7 nm, SD 0.5), periodicity of storage material was measured in renal tissue of a Fabry and Gaucher patient. Periodicity of the characteristic zebra bodies found in Fabry disease (Figure 3C) was 4.3 nm (SD 0.2), which is in line with previous findings.^38^ In contrast, glomerular Gaucher cells contain inclusion bodies that appear in microtubular arrangements (Figure 3D) which had a diameter of 60 nm.

We propose that in our patient renal disease has developed as a consequence of disease progression. Indeed over time, he displayed a chronic progressive course with an increase in spleen volume and a decrease of diffusion capacity (depicted in Figure 1). Moreover, lipid accumulation and the presence of foam cells are associated with renal injury and nephrotic range proteinuria.^39^, ^40^, ^41^, ^42^ However, clinical manifestations of renal injury are an uncommon finding in patients with the chronic visceral subtype of ASMD with a similar disease burden. We speculate that the progressive accumulation in this case has led to a pro‐inflammatory and fibrinogenic response, as seen in FD patients.^43^ Since it is unknown how frequent (mild) renal impairment or proteinuria occurs in ASMD, we suggest screening for proteinuria and plasma creatinine during routine follow‐up.

At this point, there are limited treatment options for our patient other than supportive care with statin therapy and an angiotensin receptor blocker in combination with a thiazide diuretic, which has reduced his proteinuria to 1.7 g/24 h (protein/creatinine ratio 1120 mg/g). ERT for ASMD shows promise, but effects on storage in the kidney have not been investigated. In Gaucher disease, renal clearance by ERT has been described in two papers: Santoro et al. describe the case of a patient with nephrotic syndrome consequent to Gaucher disease, who was treated with ERT which resulted in clearance of proteinuria^26^ and de Boer et al report the absence of storage in a transplanted kidney after years of ERT in a patient with a history of proteinuria and foamy macrophages in the kidney.^27^ ERT in Fabry results in clearance of Gb3 in endothelial cells, mesangial cells of the glomerulus and interstitial cells, and to a lesser extent in podocytes and distal tubular epithelium.^44^, ^45^

## 5 PATIENT CONSENT

All patients provided written consent for the use of tissue obtained for diagnostic purposes in scientific publications. The patient we describe also provided written consent for the use of his clinical data.

## CONFLICT OF INTEREST

Martijn van der Lienden, Johannes Aerts and Jan Aten have no competing interests to declare. Eline Eskes is involved as a sub‐investigator in a pre‐marketing study with Sanofi Genzyme. Joris Roelofs has had incidental consultancy agreements with Sanofi Genzyme. Liffert Vogt reports having consultancy agreements with AstraZeneca, Sanofi Genzyme and Vifor Pharma. Carla Hollak is involved in pre‐marketing studies with Sanofi Genzyme, Protalix and Idorsia.
